# Microbiome genetics underpins chemotherapy

**DOI:** 10.18632/oncotarget.22066

**Published:** 2017-10-25

**Authors:** Timothy A. Scott, Leonor M. Quintaneiro, Filipe Cabreiro

**Affiliations:** Filipe Cabreiro: Institute of Structural and Molecular Biology, University College London and Birkbeck, London, UK

**Keywords:** microbiota, 5-fluorouracil, elegans, cancer, drugs

Cytotoxic drugs are a mainstay of cancer treatment, but response rates are notoriously variable between patients. While some of this disparity is explained by host genetics, it is becoming increasingly clear that other factors are involved. A recent report from our laboratory [1] suggests that the plethora of microorganisms that inhabit our bodies, or microbiome, are important contributors to the efficacy of fluoropyrimidine-based drugs.

The mammalian microbiome is extraordinarily complex, consisting of a hundred trillion cells and thousands of species that collectively encode 5 million distinct genes. Although the idea that gut microbes can affect drug responses has existed for at least half a century, the complexity and technical limitations have made mechanistic insights difficult. Microbes may interfere with host-targeted drugs directly (metabolic transformation) and/or indirectly (regulating host metabolism and/or cellular signalling). For example, gut bacteria are capable of metabolically reactivating the chemotherapeutic CPT-11, increasing toxicity, and unexpected microbial hydrolysis of the antiviral sorivudine resulted in the death of patients undergoing fluoropyrimidine-based chemotherapy (reviewed in [2]).

Host-microbe-drug interactions are commonly studied in mice through the use of antibiotics or in a germ-free environment. Despite such studies having made important contributions to our understanding of host-microbe interactions, they are expensive, low-throughput and remain complex, making the underlying biological mechanisms difficult to unravel. The nematode *C. elegans* offers a simplified and tractable, yet evolutionary conserved, host-microbe model which addresses many of these drawbacks. Like us, worms establish a symbiotic relationship with their microbes, requiring them for optimal development and metabolic health [3].

We utilised the unique advantages of the *C. elegans* model to explore how bacteria can change the host response to the antimetabolite pro-drug 5-fluorouracil (5-FU), commonly used in the treatment of colorectal cancer (CRC) [3]. 5-FU is intracellularly converted into cytotoxic fluoropyrimidines by ribonucleotide pathways, the predominant of which (FdUMP) inhibits a key enzyme, thymidylate synthase (TS), thereby blocking the production of thymidylate and disrupting DNA replication and cellular division [4]. Given the evolutionary conservation of these pathways in prokaryotes, we postulated that microbes may be capable of modifying the host’s response to this class of drugs.

Fluoropyrimidines inhibit development, fecundity and cancer-like germline hyper-proliferation in *C. elegans*, enabling a robust and high-throughput readout of drug efficacy. We observed up to 80-fold changes in drug toxicity depending on the bacterial strain the animal was colonized with. To thoroughly define the microbial mechanism(s) behind modulation of drug efficacy on the host, we devised an innovative 3-way host-microbe-drug high-throughput screen of *C. elegans* using the Keio collection of *E. coli* single deletion mutants [1, 5]. From more than 55,000 individual conditions tested, this screen identified vitamins B6 and B9 and ribonucleotide metabolism as key contributors to 5-FU action in the host. Disruption of the bacterial B6 and B9-associated pathways (glycine cleavage system and one-carbon metabolism), which are linked to ribonucleotide metabolism via TS, reduced drug efficacy in the host. These results suggested bacterial ribonucleotide metabolism as the primary mediator of drug toxicity in *C. elegans* [1].

To mechanistically explore the bacterial mediation of 5-FU toxicity on the host, we performed *in vivo* and *in vitro* measurements together with metabolomic analysis and found that microbes have the capacity to modify and excrete activated toxic fluoronucleotides including FUMP, FUTP and FdUMP, which are ultimately responsible for the increased efficacy of drug therapy. These activated compounds, as well as the toxic effects of 5-FU, were abrogated in a genetically modified bacterial strain lacking genes encoding the enzymes of ribonucleotide salvage: *upp*, *udk*, and *udp*. Crucially, profiles of 5-FU metabolites isolated from worms were identical to profiles of the bacterial strain they were co-cultured with, indicating that bacterial metabolism dictates drug metabolism rather than that of the worm. In addition, we demonstrated that the ability for bacteria to convert 5-FU leads to reduced germline hyperproliferation and promotes survival in nematode cancer models [1].

We also identified a secondary mechanism by which bacteria are able to manipulate fluoropyrimidine efficacy in *C. elegans*. We found bacteria deficient in the enzyme nucleoside diphosphate kinase (*ndk*) increased drug toxicity on the host via a mechanism independent of the accumulation of modified fluoronucleotides. Rather, metabolomic profiling revealed that disruption of bacterial *ndk* results in deoxynucleotide imbalance, which sensitises the host to 5-FU-induced DNA damage through amplified autophagy. Similarly, the gut microbe *Fusobacterium nucleatum* promotes resistance to combinatorial chemotherapy of 5-FU and Oxaliplatin in CRC patients by modulating autophagy [6]. Altogether, these studies demonstrate that microbes regulate chemotherapy by direct biochemical conversion of toxic chemotherapy pro-drugs [1, 5] or indirectly by regulating the tumour microenvironment [1, 6].

The power of understanding the mechanisms behind microbial manipulation of chemotherapy and other drugs is difficult to exaggerate. The ability to tweak a living system and “pull the strings” by a variety of interventions holds great potential as an avenue for personalised medicine. Routine profiling of patient microbiome composition, host and microbiome genetics and disease status, could lead to predictive models to ensure optimal treatment outcomes. Therapeutics could be designed to directly target the microbiota, or patients colonised with ‘designer microbes’, to ensure optimal drug activity.

It is almost certain that the currently reported host-microbe-drug interactions are only the tip of the iceberg, and that there are many exciting times ahead in our continued comprehension of how bugs modulate drugs.
